# Commentary: Analysis of SUMO1-conjugation at synapses

**DOI:** 10.3389/fncel.2017.00345

**Published:** 2017-10-30

**Authors:** Kevin A. Wilkinson, Stéphane Martin, Shiva K. Tyagarajan, Ottavio Arancio, Tim J. Craig, Chun Guo, Paul E. Fraser, Steven A. N. Goldstein, Jeremy M. Henley

**Affiliations:** ^1^School of Biochemistry, Centre for Synaptic Plasticity, University of Bristol, Bristol, United Kingdom; ^2^Centre National de la Recherche Scientifique, Institut National de la Santé et de la Recherche Médicale, IPMC, Université Côte d'Azur, Nice, France; ^3^Institute of Pharmacology and Toxicology, University of Zurich, Zurich, Switzerland; ^4^Taub Institute and Department of Pathology and Cell Biology, Columbia University, New York, NY, United States; ^5^Department of Applied Sciences, University of the West of England, Bristol, United Kingdom; ^6^Department of Biomedical Science, University of Sheffield, Sheffield, United Kingdom; ^7^Tanz Centre for Research in Neurodegenerative Diseases, University of Toronto, Toronto, ON, Canada; ^8^Stritch School of Medicine, Loyola University, Chicago, IL, United States

**Keywords:** SUMO1, synapse, sumoylation, neurons, post-translational modification

There is a large and growing literature on protein SUMOylation in neurons and other cell types. While there is a consensus that most protein SUMOylation occurs within the nucleus, SUMOylation of many classes of extranuclear proteins has been identified and, importantly, functionally validated. Notably, in neurons these include neurotransmitter receptors, transporters, sodium and potassium channels, mitochondrial proteins, and numerous key pre- and post-synaptic proteins (for reviews see Martin et al., 2007b; Scheschonka et al., 2007; Craig and Henley, 2012; Luo et al., 2013; Guo and Henley, 2014; Henley et al., 2014; Wasik and Filipek, 2014; Peng et al., 2016; Schorova and Martin, 2016; Wu et al., 2016). Furthermore, several groups have reported SUMO1-ylated proteins in synaptic fractions using biochemical subcellular fractionation approaches, using a range of different validated anti-SUMO1 antibodies (Martin et al., 2007a; Feligioni et al., 2009; Loriol et al., 2012; Luo et al., 2013; Marcelli et al., 2017) and many studies have independently observed colocalization of SUMO1 immunoreactivity with synaptic markers (Martin et al., 2007a; Konopacki et al., 2011; Gwizdek et al., 2013; Jaafari et al., 2013; Hasegawa et al., 2014; Ghosh et al., 2016). Tirard and co-workers (Daniel et al., 2017) directly challenge this wealth of compelling evidence. Primarily using a His6-HA-SUMO1 knock-in (KI) mouse, the authors contest any significant involvement of post-translational modification by SUMO1 in the function of synaptic proteins.

## On what basis do Daniel et al. argue against synaptic SUMOylation?

Most of the experiments reported by Daniel et al. use a knock-in mouse that expresses His6-HA-SUMO1 in place of endogenous SUMO1. Using tissue from these mice, followed by immunoprecipitation experiments, they fail to biochemically identify SUMOylation of the previously validated SUMO targets synapsin1a (Tang et al., 2015), gephyrin (Ghosh et al., 2016), GluK2 (Martin et al., 2007a; Konopacki et al., 2011; Chamberlain et al., 2012; Zhu et al., 2012), syntaxin1a (Craig et al., 2015), RIM1α (Girach et al., 2013), mGluR7 (Wilkinson and Henley, 2011; Choi et al., 2016), and synaptotagmin1 (Matsuzaki et al., 2015). Moreover, by staining and subcellular fractionation, they also fail to detect protein SUMOylation in synaptic fractions or colocalization of specific anti-SUMO1 signal with synaptic markers. On this basis, they conclude there is essentially no functionally relevant SUMO1-ylation of synaptic proteins.

## What are the reasons for these discrepancies?

### Inefficiency of His6-HA-SUMO1 conjugation and compensation by SUMO-2/3

A major cause for concern is that there is 20–30% less SUMO1-ylation in His6-HA-SUMO1 KI mice than in wild-type (WT) mice (Tirard et al., 2012; Daniel et al., 2017). Moreover, in the paper initially characterizing these KI mice, Tirard et al. showed that while total protein SUMO1-ylation is reduced, total SUMO2/3-ylation is correspondingly increased (Tirard et al., 2012). Thus, His6-HA-SUMO1 conjugation is significantly impaired and most likely compensated for by increased conjugation by SUMO2/3. Crucially, however, Daniel et al. do not examine modification by SUMO2/3 at any point in their recent study.

Given that SUMO modification is notoriously difficult to detect, the 20–30% reduction in His6-HA-SUMO1 compared to wild-type SUMO1 conjugation will make it even more technically challenging. Moreover, this deficit in SUMO1-ylation may well be offset by an increase in SUMO2/3-ylation of individual proteins, but this likely compensation was not tested. Because these deficits alone could explain why Daniel et al. failed to detect SUMO1 modification of the previously characterized synaptic substrate proteins, it is surprising that they did not attempt to recapitulate the SUMO1-ylation of the target proteins under the endogenous conditions in wild-type systems used in the original papers since this approach would circumvent potential issues of ineffective conjugation or localization of the His6-HA-SUMO1.

### Lack of functional studies on the substrates they examine

Daniel et al. confine their studies to immunoblotting and immunolabeling. However, these techniques address only one aspect of validating a *bone fide* SUMO substrate. It is at least as important to examine the effects of target protein SUMOylation in functional assays. Function-based approaches such as electrophysiology or neurotransmitter release assays are not reported or even discussed by Daniel et al. This is an extremely important omission. We argue that simply because SUMO1-ylation of a protein is beneath the detection sensitivity in a model system that exhibits sub-endogenous levels of SUMO1-ylation, does not mean that protein is not a functionally important and physiologically relevant SUMO1 substrate.

### Insensitivity or inadequate use of assay systems

#### Failure to detect GluK2 SUMOylation

GluK2 is a prototypic synaptic SUMO1 substrate that has been validated in exogenous expression systems, neuronal cultures, and rat brain (Martin et al., 2007a; Konopacki et al., 2011; Chamberlain et al., 2012; Zhu et al., 2012).

Daniel et al. attempt to detect SUMOylation of GluK2 using immunoprecipitation experiments from the His6-HA-SUMO1 KI mice. However, a key flaw in this experiment is that the C-terminal anti-GluK2 monoclonal rabbit antibody used does not recognize SUMOylated GluK2 because its epitope is masked by SUMO conjugation. Thus, due to technical reasons, the experiment shown could not possibly detect SUMOylated GluK2 whether or not it occurs in the KI mice.

#### Subcellular fractionation and immunolabeling

Daniel et al. perform subcellular fractionation and anti-SUMO1 Western blots to compare His6-HA-SUMO1 KI and SUMO1 knockout (KO) mice. In the KI mice they fail to detect SUMO1-ylated proteins in synaptic fractions. Importantly, however, they do not address what happens in WT mice, which, unlike the KI mice, exhibit normal levels of SUMO1-ylation.

While the authors provide beautiful images of SUMO1 immunolabeling in neurons cultured from WT, His6-HA-SUMO1 KI mice, and SUMO1 KO mice, in stark contrast to previous reports using rat cultures (Martin et al., 2007a; Konopacki et al., 2011; Gwizdek et al., 2013; Jaafari et al., 2013), they detect no specific synaptic SUMO1 immunoreactivity in neurons prepared from WT mice. We note, however, that the nuclear SUMO1 staining in neurons from His6-HA-SUMO1 KI mice is weak, and even weaker in WT neurons. Given that a very large proportion of SUMO1 staining is nuclear, these low detection levels would almost certainly rule out visualization of the far less abundant, but nonetheless functionally important, extranuclear SUMO1 immunoreactivity.

## In conclusion

Given these caveats, we suggest that the failure of Daniel et al. to detect synaptic protein SUMO1-ylation in His6-HA-SUMO1 KI mice is due to intrinsic deficiencies in this model system that prevent it from reporting the low, yet physiologically relevant, levels of synaptic protein modification by endogenous SUMO1. In consequence, we question the conclusions reached and the usefulness of this model for investigation of previously identified and novel SUMO1 substrates.

## Author contributions

KW and JH wrote the first draft. All other authors contributed ideas, writing and creative input into the generation of the final manuscript.

### Conflict of interest statement

The authors declare that the research was conducted in the absence of any commercial or financial relationships that could be construed as a potential conflict of interest.
